# Moderate Intensity Resistive Training Reduces Oxidative Stress and Improves Muscle Mass and Function in Older Individuals

**DOI:** 10.3390/antiox8100431

**Published:** 2019-09-26

**Authors:** Alessandra Vezzoli, Simona Mrakic-Sposta, Michela Montorsi, Simone Porcelli, Paola Vago, Ferdinando Cereda, Stefano Longo, Marcello Maggio, Marco Narici

**Affiliations:** 1 Institute of Clinical Physiology, National Research Council (CNR), ASST Grande Ospedale Metropolitano Niguarda, 20121 Milan, Italy; alessandra.vezzoli@cnr.it (A.V.); simona.mrakicsposta@cnr.it (S.M.-S.); 2 Department of Human Sciences and Promotion of the Quality of Life, San Raffaele Roma Open University, 20121 Milan, Italy; michela.montorsi@uniroma5.it; 3 Institute of Biomedical Technologies, National Research Council (CNR), Segrate, 20121 Milan, Italy; simone.porcelli@itb.cnr.it; 4 Interfaculty of Education and Medicine, Università Cattolica del Sacro Cuore, 20121 Milan, Italy; paola.vago@unicatt.it (P.V.); ferdinando.cereda@unicatt.it (F.C.); 5 Department of Biomedical Sciences for Health, Università degli Studi di Milano, 20133 Milan, Italy; stefano.longo@unimi.it; 6 Department of Clinical and Experimental Medicine, University of Parma, 43126 Parma, Italy; marcellogiuseppe.maggio@unipr.it; 7 Department of Biomedical Sciences, University of Padua, 35122 Padua, Italy

**Keywords:** resistive training, muscle mass, muscle strength, oxidative stress

## Abstract

An innovative moderate-intensity resistive exercise-training (RT) program was tested in thirty-five sarcopenic elders (SAR). The subjects were randomized into two groups: SAR training (SAR-RT), n = 20, 73.0 ± 5.5 years, or SAR non-training (SAR-NT), n = 15, 71.7 ± 3.4 years. The training consisted of 12-week progressive RT, thrice/week, at 60% one-repetition maximum (1RM), 3 sets, 14–16 repetitions for both upper and lower limbs. The pre and post intervention measurements included: the skeletal muscle index (SMI%); strength (1RM); stair-climbing power (SCP); muscle thickness (MT) of vastus lateralis (VL) and elbow flexors (EF), VL pennation angle (PA), rectus femoris (RF) anatomical cross-sectional area (ACSA); reactive oxygen species (ROS), total antioxidant capacity (TAC), protein carbonyls (PC), thiobarbituric acid-reactive substances (TBARS), 8-isoprostane (8-iso-PGF2-α), 8-OH-2-deoxyguanosine (8-OH-dG), as markers of oxidative stress/damage (OxS). In SAR-RT, SCP increased by 7.7% (*P* < 0.01), MT increased by 5.5% for VL, 10.4% for EF and PA increased by 13.4% for VL (*P* < 0.001 for all). The RF ACSA increased by 14.5% (*P* < 0.001). 1RM significantly increased by at least 67% for all muscles tested. Notably muscle strength (1RM) positively correlated (*P* < 0.001) with TAC and negatively with PC (*P* < 0.001). In conclusion, moderate intensity RT is an effective strategy to increase muscle mass and strength in SAR, while minimizing OxS.

## 1. Introduction

Aging entails a decline in muscle mass, function and mobility. Even in healthy individuals, muscles become smaller, weaker and less powerful as age progresses, impairing their ability to perform essential physical activities of daily life, while increasing the risk of falls and prevalence of multimorbidity [1,2,3,4,5]. Several factors are believed to cause this age-related loss of muscle mass and function, known as sarcopenia [6,7]: (1) neuro-endocrine changes, (2) oxidative stress (OxS), (3) inflammation, (4) nutritional/protein metabolism changes, (5) and reduced physical activity [6,7,8]. Among these factors, oxidative damage induced by mitochondrial dysfunction has been recognized as a major factor contributing to the age-dependent muscle degeneration [9] through α-motoneuron degeneration and damage to the neuromuscular junction [9], as well as the reduction in satellite cell number and function in old age [10,11]. Moreover, it has been proposed that damage to mitochondrial DNA (mtDNA) plays a significant role in muscle aging because genetic insults to mtDNA may lead to mitochondrial dysfunction and generate a vicious-cycle of increased OxS [12].

Despite the significant loss of muscle mass and strength (approximately 30% of muscle mass and 45% of muscle strength are lost between 20 and 80 years of age) [13], a substantial mitigation of sarcopenia may be achieved by resistance exercise training (RT) programs. Indeed, a wealth of studies has shown that RT is effective for recovering muscle mass, strength and physical performance in older individuals [14], even in the oldest old [15] as defined by the WHO [16]. Muscle hypertrophy produced by RT programs is mostly achieved through the activation of the AKT-mTOR protein signaling pathways leading to an increase in protein synthesis and a decrease in protein breakdown by an AKT-mediated inhibition of FOXO [17]. Further, a RT-mediated reduction of inflammation [18] and of oxidative stress [19] may also contribute to the gain in muscle mass. However, senescent muscle seems to be more susceptible to OxS during exercise due to the age-related ultrastructural and biochemical changes that facilitate ROS formation [20]. Furthermore, muscle repair and regeneration capacities are reduced in old age and this could potentially enhance the accrual of cellular oxidative damage [21,22,23,24]. Therefore, it seems likely that exercise, especially if strenuous, may be associated with an abnormal production of ROS, leading to endogenous antioxidants reduction, eventually damaging biological molecules and key cellular components. Recently, it has been proposed, despite some controversy, that moderate intensity resistive training programs (3–5 × 10 repetitions at 50–80% strength (1RM)) may improve antioxidant defenses in the elderly [25]. The use of moderate intensity RT seems also advocated by other studies suggesting that low to medium intensity RT programs may prove useful for combating sarcopenia [26,27]. These benefits are related to the findings that low intensity/high volume RT seems particularly effective for counteracting the anabolic resistance of ageing [28,29] and that moderate intensity and volume RT (3 × 9 repetitions at 60% 1RM) maximizes myofibrillar protein synthesis in the elderly [30].

Hence, this study aimed at testing the hypothesis that a moderate-intensity RT program in older individuals would be effective in combating sarcopenia by increasing muscle mass and strength, while minimizing oxidative stress balance perturbation. The main novel approach of this study was that of relating changes in oxidative stress and in particular of ROS, to skeletal muscle morphological and functional responses to moderate RT in the same population of older individuals.

## 2. Methods

### 2.1. Participants

Sixty-two old (over 65 years) independent, community dwelling individuals were recruited for the study. The exclusion criteria, based on the Medically Stable definition [31], were the presence of: (i) cardiovascular, neurologic and metabolic diseases, severe airways obstruction, resting systolic blood pressure > 160 mmHg or diastolic blood pressure > 100 mmHg; (ii) severe emotional disturbance, mental illness or depression in the past two years; (iii) debilitating arthrosis of the lower limbs (classified as inability to perform maximum contractions of the lower limbs muscles without pain), fractures of the lower limbs in the last two years, fractures on the upper limbs in the past six months, arthroscopic surgery of lower limb joints in the past two years, any cause of loss of mobility for a period greater than one week in the past two months or greater than two weeks in the last six months; (iv) the daily intake of painkillers. The participants were recreationally active, and none were practicing sport at a competitive level, or engaged in any regular high-intensity physical activity program. Further, all were non-smokers. For all the experimental phases, they refrained from alcohol consumption and were asked to avoid any form of exercise for 24 h before basal testing. All participants signed written informed consent after being advised on all procedures and purposes of the study. The procedures were in accordance with the Declaration of Helsinki, and Ethics approval (IBFM-3-43-06092017) by the relevant Institutional Review Boards was obtained for this study.

### 2.2. Study Design

**Figure 1:**
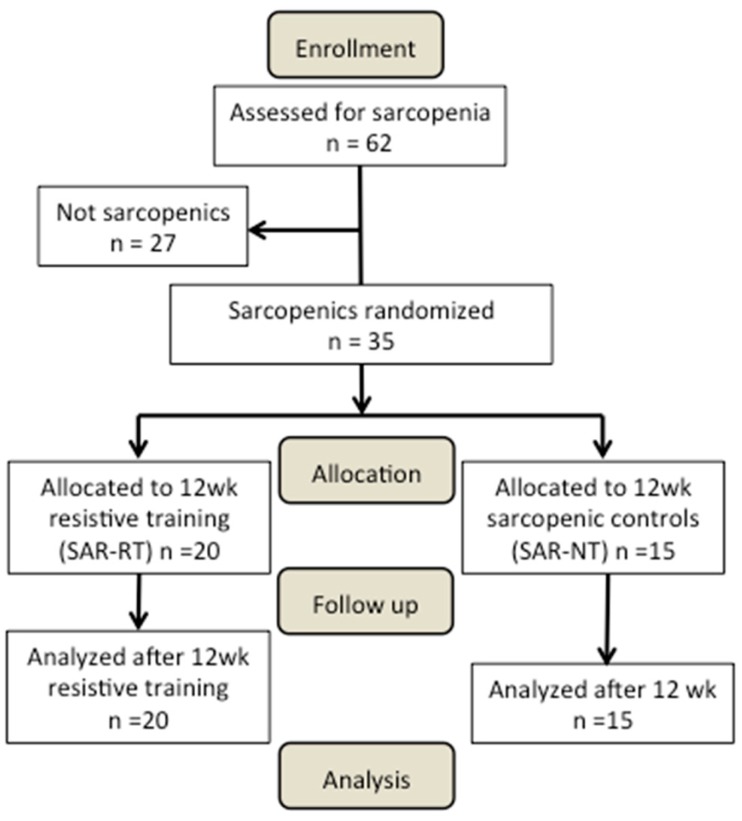
Participant flow through the study.

At the first study visit (Figure 1), the participants were screened for sarcopenia on the basis of their skeletal muscle mass (see Sarcopenia diagnosis section for further details). After completion of the medical screening evaluations, 35 participants were deemed to be sarcopenic (SAR, n = 35). The criteria for being included in the SAR group were: (i) being classified as class I or II sarcopenic (see below for further details); (ii) having a BMI between 20 and 29.9 kg·m^−2^; (iii) having a willingness to comply with the protocol, including maintaining eating habits, and demonstrating the ability to take part in muscle strength and physical function assessments. Thereafter, the SAR participants were randomly divided into two groups: A sarcopenic group undergoing a 12-week RT program (SAR-RT, n = 20); a non-exercising sarcopenic group (SAR-NT n = 15) acting as the control.

The body composition, functional performance and OxS markers from biological samples were evaluated. The SAR-RT and SAR-NT were tested twice: before (pre) and after (post) the intervention. During this period, all participants were encouraged to maintain their regular activities of daily living and their nutritional habits. All participants were familiarized with each evaluation involved in this investigation.

### 2.3. Sarcopenia Diagnosis

The participants were diagnosed as sarcopenic based on their skeletal muscle mass [1] estimated by the bioelectrical impedance analysis (BIA), which was expressed as the skeletal muscle mass index (SMI = (skeletal muscle mass/body mass) × 100). The participants were considered to have a normal SMI if it was greater than one standard deviation above the sex-specific mean for young adults (aged 18–39). Class I sarcopenia was considered present in the participants whose SMI was within one to two standard deviations of young adult values, and class II sarcopenia was present in the participants whose SMI was below two standard deviations of young adult values [1]. To simplify these ranges, the final cut-off levels for normal SMI, class I, and class II sarcopenia were set as follows: men greater than 37%, 37% to 31%, and less than 31%, respectively; women greater than 28%, 28% to 22%, and less than 22%, respectively [1]. Thirty-five participants fulfilled the inclusion criteria of either type I or II sarcopenia. Thereafter, they were randomly assigned to the SAR-RT (n = 20, 10 females and 10 males) or SAR-NT (n = 15, 9 females and 6 males) groups. The choice for a higher number of participants in the SAR-RT group was to ensure sufficient statistical power and maintain an adequate number in case of dropouts. All the participants’ characteristics are provided in Table 1.

### 2.4. Training Intervention

The participants of the SAR-RT group trained 3 times per week for 12 weeks. The RT protocol started with a 6–8 min aerobic warm-up (treadmill or bike at level 1, depending on the preference of the subject), followed by 3 series of 14–16 repetitions of chest press, horizontal leg-press, vertical row, and shoulder exercises with free weights (lateral raise) exercises at 60% 1RM. This load intensity was purposely chosen as it has been found to correspond to the relative load at which myofibrillar fractional rate of protein synthesis reaches a peak in older individuals [30]. The 1RM was re-tested every week to maintain training intensity at 60% 1RM with 14–16 repetitions. A one-minute rest interval between each series and muscle group was allowed. All the participants were individually supervised by physical education professionals throughout each training session to ensure subject safety and adherence to the training protocol. The participants were requested not to perform any other type of physical exercise (except for their regular activities of daily living) during the entire training period.

### 2.5. Body Composition

Body composition was assessed using a BIA 101 bioimpedance device (Akern Srl, Florence, Italy), which applies an 800-µA current at a frequency of 50 KHz. The values of reactance, resistance, basal metabolism rate, phase angle, extracellular water, and body cellular mass were computed to obtain estimates of fat free mass (kg and %), fat mass (kg and %), and muscle mass (kg and %). Among these values, skeletal muscle mass was used for the calculation of the SMI according to Janssen et al. [1]. The validity of BIA has been demonstrated previously [32].

### 2.6. Functional Performance Evaluations

The following evaluations were performed for assessing functional performance: short physical performance battery (SPPB) [32], handgrip, “get up and go” and stair climbing tests.

The SPPB includes three tests of physical function: balance, gait speed, and chair stands [33]. It has been used as a predictive tool for possible disability and can aid in the monitoring of function in older people. Each component is scored from 0 (worst performance) to 4 (best performance) points. The final score is the sum of the three tests, up to 12 points. The SPPB has been shown to have predictive validity showing a gradient of risk for mortality, nursing home admission, and disability [34]. For balance, the participants were standing barefoot facing a wall, placing the heel of the preferred foot on the side of the big toe of the other one. The time to stand in this position without moving the feet was recorded up to 10 s. The participants who were unable to accomplish the task were assessed standing with the feet side-by-side, whereas those ones which completed the task were further assessed in the full tandem position (heel of the preferred foot in front and in contact with the toes of the other foot) for 10 s in both cases. The participants were allowed to maintain balance moving the upper limbs and swinging the body during the tasks, if necessary. For gait speed, the ability to walk in a 4-m path in the absence of obstacles, with the possibility to perform two additional steps at the end was assessed. The participants walked at their usual speed to the far end of the path as if they were walking down the street to go to the supermarket. The test was repeated twice with 30 s of rest in between the trials, and the shortest recorded time was used for the analysis. For lower limb strength, the participants were asked to sit down and get up from a normal office chair (43–45 cm) five times as fast as possible with the arms crossed at the chest, with feet adherent to the floor and controlling the descent. The time was stopped when the participant was at the end of the fifth ascending phase. The participants were barefoot and the test was carried out twice interspersed by 1 min 30 s of rest, with the best performance considered for the analysis.

Hand grip test. This test consisted of measuring handgrip muscle force by a dynamometer (JAMAR PLUS +, Sammors Preston, Rolyon, Bolingbrook, IL, USA). The participants were placed in the upright position, the upper limbs along the sides, the legs slightly apart, and were asked to tighten the dynamometer as strong as possible for three seconds. The test was repeated three times for each hand.

Get up and go test. The participants were sitting on a chair (43–45 cm high) located 3 m from a wall leaning against the backrest. At the operator signal, they had to get up, walk towards the wall without touching it, turn around, go back to the chair and sit down. If necessary, the armrests were used during the get up phase. The participants were asked to perform this sequence as quickly as possible without running. The test was executed twice with 1-min rest in between and the best performance was considered for the analysis.

Stair climbing. This test was performed on a 12-steps staircase, each step 16 cm high. The participants started from a 60-cm wide platform and climbed the stairs as quick as possible. No step could be skipped and the handrail could be used if necessary. The stopwatch started when the first foot touched the first step and was stopped at the 12th step. Stair climbing was performed twice with 1 min 30 s of rest in between. The best time to complete the task was used for the analysis.

### 2.7. Muscle Morphology

The participants laid supine on an examination couch with the knee almost fully extended (150° of extension, with 180° being fully extension) and the upper limbs in anatomical position. B-mode ultrasonography (M-Turbo, SonoSite, Bothell, WA, USA) fitted with a linear-array probe (5 cm, 7 MHz) was used for obtaining images in vivo at rest at different muscle locations of the upper and lower right limbs. The probe was held perpendicular to the skin surface by an expert operator, which ensured minimal pressure was applied to the muscle belly examined. The 50% of femur length from the greater trochanter to the patella was used as a scanning site for the vastus lateralis (VL) and rectus femoris (RF) muscles [35,36]. The two-thirds of the distance from the acromion to the antecubital crease was chosen for the elbow flexors (EF) [37]. For the VL and EF, the images were obtained along their respective mid-sagittal planes, which included both superficial and deep aponeuroses and a number of clearly visible fascicles for VL. For the RF, the images were taken along its short axis, to include clearly the anatomical cross-sectional area (ACSA) of the muscle. Three images were taken at each site for reliability purposes and to minimize intra-operator variability, and were analyzed offline by an expert operator using the open source Image J software (NIH, Bethesda, MD, USA).

**Figure 2:**
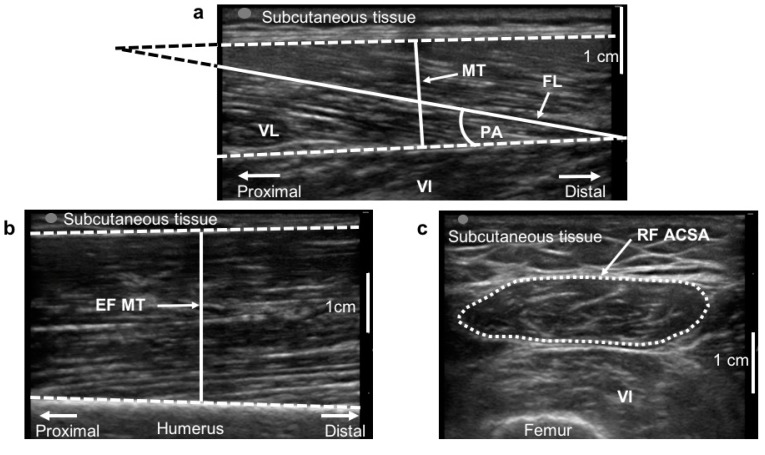
Ultrasound images analysis. Ultrasound images analysis for vastus lateralis (VL, **a**), elbow flexors (EF, **b**) and rectus femoris (RF, **c**) muscles. (**a**): muscle thickness (MT), fascicle length (FL) and pennation angle (PA) are highlighted. The non-visible part of FL was extrapolated as shown. (**b**): elbow flexors MT. (**c**): the anatomical cross-sectional area (ACSA) of RF is represented with a dotted line. VI, vastus intermedius.

For the VL, muscle thickness (MT), defined as the distance between the deep and the superficial aponeurosis, and pennation angle (PA), defined as the angle of muscle fascicles with the deep aponeurosis, were measured (Figure 2a). For the EF (Figure 2b), only MT was measured, while for the RF (Figure 2c) muscle ACSA was calculated after digitization of the muscle perimeter using the imaging software. All morphological parameters were measured pre and post-12 weeks in both the SAR-RT and SAR-NT.

### 2.8. Muscle Strength (1RM)

During the familiarization session, the correct exercise execution and timing of the concentric and eccentric phase were explained and practiced using submaximal and near-maximal loads. After at least four days, 1RM strength was estimated for the chest press, horizontal leg-press, vertical row, and lateral raise exercises. The large muscle group assessments were performed before those involving the smaller muscle groups. To facilitate recovery, exercises were alternated between the upper (chest press and vertical row) and lower body (leg press). After a warm-up with a light load (allowing 10–12 repetitions), the load was gradually increased until the 1RM was reached within 3–6 attempts. The rest periods of 2 min were introduced between attempts, and of 5 min between exercises.

### 2.9. Biological Samples

Each sarcopenic participant visited the laboratory before and immediately after 12 weeks for blood and urine collections. Approximately 5 mL of blood was drawn from the antecubital vein. The blood samples were collected in heparinized vacutainer tubes (Becton Dickinson and Company, Oxford, UK), and plasma was separated by centrifuge (5702R, Eppendorf, Hamburg, Germany) at 3000× *g* for 5 min at 4 °C. The samples of plasma were then immediately stored in multiple aliquots at −80 °C until the analyses, which were performed within two weeks from collection. In addition, 50 µL of capillary blood was taken from the fingertip and collected in heparinized capillary tubes (Cholestech LDX, Mannheim, Germany). The aliquots of the urine were stored at −80 °C until the analyses were performed.

### 2.10. Analytical Procedures

EPR Measurements. An X-band EPR instrument (E-scan-Bruker BioSpin GmbH, Billerica, MA, USA) was adopted for ROS production determination. The instrument allowed the use of low-concentration amounts of paramagnetic species in small (50 µL) samples. ROS half-life is too short if compared to the EPR time scale so that they are EPR-invisible but become EPR detectable once trapped with specific compounds (probe) and transformed into more stable radical species. For each recruited subject, the ROS production rate was determined at rest by means of a recently implemented EPR method [38,39] analyzing 50 µL capillary blood samples treated with CMH (1-hydroxy-3-methoxycarbonyl-2,2,5,5-tetramethylpyrrolidine) probe solution (1:1). 50 µL of the obtained solution was put in a glass EPR capillary tube (Noxygen Science Transfer & Diagnostics, Elzach, Germany) that was placed inside the cavity of the E-scan spectrometer for data acquisition. The acquisition parameters were the following: microwave frequency 9.652 GHz; modulation frequency 86 kHz; modulation amplitude 2.28 G; sweep width 60 G; microwave power 21.90 mW; number of scans 10; and receiver gain 3.17 × 10^1^. The sample temperature was firstly stabilized and then kept at 37 °C by the Temperature and Gas Controller Bio III unit, interfaced to the spectrometer. The spectra were recorded and analyzed by using Win EPR software (2.11 version) supplied by Bruker. The EPR measurements allowed the attainment of a relative quantitative determination of ROS production rate in the collected samples. Then, all data were converted to absolute concentration levels (µmol·min^−1^) by adopting CP∙(3-Carboxy-2,2,5,5-tetramethyl-1-pyrrolidinyloxy) stable radical as an external reference.

Total antioxidant capacity (TAC). Plasma TAC was measured by an enzymatic kit (Cayman Chemical, Ann Arbor, MI, USA). This assay is based on the ability of plasma antioxidants to inhibit the oxidation of 2,2′-azinobis (3-ethylbenzithiazoline) sulfonic acid (ABTS) to the radical cation ABTS^+^ by a peroxidase. The amount of the produced ABTS^+^ is assessed by measuring the absorbance signals at 750 nm. The antioxidants concentration is proportional to the suppression of the absorbance signal. The TAC was evaluated by a trolox (6-hydroxy-2,5,7,8-tetramethylchroman-2-carboxylic acid) standard curve and was expressed as trolox-equivalent antioxidant capacity concentration (mM).

Protein Carbonyls (PC). The accumulation of oxidized proteins was assessed by measuring the content of reactive carbonyls. A Protein Carbonyl assay kit (Cayman Chemical, Ann Arbor, MI, USA) was used to evaluate colorimetrically-oxidized proteins. The samples were read at 370 nm as described in detail by the manufacturer. The oxidized protein values obtained were normalized to the total protein concentration in the final pellet (absorbance reading at 280 nm), in order to consider the protein loss during the washing steps, as suggested in the user manual.

Thiobarbituric acid-reactive substances (TBARS). The measurement of TBARS is a utilized method to detect lipid peroxidation. The TBARS assay kit (Cayman Chemical, Ann Arbor, MI, USA) was used which allows a rapid photometric detection of the thiobarbituric acid malondialdehyde (TBAMDA) adduct at 532 nm. A linear calibration curve was computed from pure malondialdehyde-containing reactions.

8-isoprostane (8-iso-PGF2-α). A competitive immunoassay was used for the determination of 8-isoprostanes, the markers of lipid peroxidation, in urine (Cayman Chemical, Ann Arbor, MI, USA). The urine was purified using the solid phase extraction cartridges. The purification and the subsequent ELISA assay were performed following the manufacturer’s recommendations. The samples and standards were read on an ELISA plate at 405-nm wavelength.

8-OH-2-deoxyguanosine (8-OH-dG). 8-OH-2-deoxyguanosine (8-OH-dG) has been established as a marker of oxidative DNA damage. This compound was quantified in excreted urine. A commercially-available enzyme immunoassay EIA kit (Cayman Chemical, Ann Arbor, MI, USA) for the measurement of 8-OH-dG was utilized. The sample 8-OH-dG concentration was determined using an 8-OH-dG standard curve.

Creatinine. The urinary concentrations of 8-iso-PGF2-α and 8-OH-dG, as any urinary marker, vary considerably in relation to renal function and the urinary parameters are usually standardized based on the amount of creatinine excreted in the urine when the collection of the 24 h urine is not possible. Thus, the urinary creatinine levels were measured by a creatinine assay kit (Cayman Chemical, Ann Arbor, MI, USA) and creatinine concentration was determined using a creatinine standard curve.

All the above samples and standards were read by a microplate reader spectrophotometer (Infinite M200, Tecam, Austria). The determinations were assessed in duplicate and the inter-assay coefficient of variation was in the range indicated by the manufacturer.

### 2.11. Statistical Analysis

The statistical analysis was performed using GraphPad Prism package (GraphPad Prism 8.0, Software Inc., San Diego, CA, USA) and Statistical Package for the Social Science (IBM SPSS Statistics v. 22, Armonk, NY, USA) software. The data were expressed as the mean ± standard deviation (SD). The normality of the data distribution was tested with the Shapiro Wilk’s test. The data were normally distributed. The prospective calculation of the sample size for SAR-RT and SAR-NT was determined choosing the 1-RM as primary outcome (GPower 3.1 [40]). For a mixed-model analysis of variance (2 × 2 ANOVA), selecting an alpha level of 0.05 and a power value of 0.80, the minimum required sample size was 12 subjects for each group. A lager population (20 SAR-RT and 15 SAR-NT) was recruited to account for potential dropouts. For the comparisons between SAR-RT and SAR-NT, a mixed-model (2 × 2) ANOVA was employed, with time (2 levels) and group (2 levels) as within and between factors, respectively. Bonferroni’s post-hoc test was applied for pairwise comparisons. The paired Student’s t-tests were used to compare muscle strength pre and post-training in SAR-RT. The Pearson’s product moment test was used to check for possible correlations between TAC, PC and strength measurements. The determination coefficient (R^2^) was also calculated. The reliability of muscle morphological measurements (MT, PA and ACSA) was tested using a two-way, a mixed model intraclass correlation coefficient (ICC, as an index of relative reliability); the standard error of measurements calculation as a percentage (SEM%, as an index of absolute reliability) assessed intra-operator reliability. The ICC values were provided with a 95% of confidence interval (95% C.I.) and considered as very high if > 0.90, high if between 0.70 and 0.89 and moderate if between 0.50 and 0.69 [39]. The sensitivity of the different parameters in detecting the changes induced by RT was checked by calculating the minimum detectable change at 95% confidence as a percentage (MDC_95_%). The significance was set with *P* < 0.05.

## 3. Results

### 3.1. Sarcopenia Diagnosis

The analysis of the SMI% values (Table 1) in SAR-RT revealed that pre-training 14 (70%) individuals were classified as type I and 6 (30%) as type II sarcopenic. After the 12-weeks RT, the prevalence of sarcopenia decreased by 15% with respect to pre-training: 3 participants were classified as type II sarcopenic, 14 as type I and 3 as non-sarcopenic. Concerning SAR-NT, 14 (93%) individuals were classified as type I sarcopenic and 1 (7%) as type II sarcopenic. After 12 weeks, the prevalence of sarcopenia in this group did not vary (Table 1).

### 3.2. Body Composition and Functional Performance

No significant differences in the skeletal muscle mass, SPPB score, handgrip and get up and go tests (Table 1) were found among the groups either at baseline or after 12 weeks (*P* > 0.05). No significant differences in stair climbing power was present among the groups at baseline. However, SAR-RT participants increased stair climbing power by 7.7% (*P* < 0.01) after the training intervention.

### 3.3. Muscle Morphology

The muscle morphology measurements presented very high reliability (ICC > 0.90) and low SEM% values. The ICC (95% C.I.) were: 0.95 (0.86–0.98) for VL PA; 0.99 (0.97–0.99) for VL MT; 0.98 (0.95–0.99) for RF ACSA, and 0.98 (0.96–0.99) for EF MT. The SEM% values were: 3.5% for VL PA; 1.8% for VL MT; 3.6% for RF ACSA; and 3.0% for EF MT.

All parameters were not significantly different at baseline (*P* > 0.05). Concerning MT and ACSA, the ANOVA revealed significant effects of time and interaction (both *P* < 0.01) in VL and EF MT, and RF ACSA. The pairwise comparisons showed significant increments in the SAR-RT group (VL MT: 5.5%, *P* < 0.001; EF MT: 10.4%, *P* < 0.001; RF ACSA: 14.5%, *P* < 0.001). No previous parameters changed significantly in the SAR-NT group (*P* > 0.05). Concerning VL PA, the ANOVA revealed the significant effect of time (*P* < 0.05) and interaction (*P* < 0.01). The pairwise comparison showed that RT induced a significant increase in PA (13.4%, *P* < 0.001) in SAR-RT. In SAR-NT, PA did not change significantly (*P* > 0.05). All results are presented in Table 2. For all investigated parameters, all increments (%) in the SAR-RT group exceeded the MDC_95_%. 

### 3.4. Muscle Strength

The 1RM tests presented very high reliability (ICC > 0.90). Specifically, ICC (95% CI) were: 0.99 (0.98–1.00) for chest press; 0.99 (0.98–0.99) for horizontal leg press; 0.99 (0.99–0.99) for vertical row; and 0.94 (0.86–0.97) lateral raise. The SEM% values were: 5.4% for chest press, 3.4% for horizontal leg press, 5.6% for vertical row, and 14.2% for lateral raise. A highly significant increase of muscle strength (1RM) of all the trained muscle groups, ranging from 66.7% to 101%, was found after the intervention (Table 3). On average, the increase in muscle strength was 85.6% after the 12-weeks RT program. All increments (%) exceeded the MDC_95_%.

### 3.5. Biological Samples

**Figure 3:**
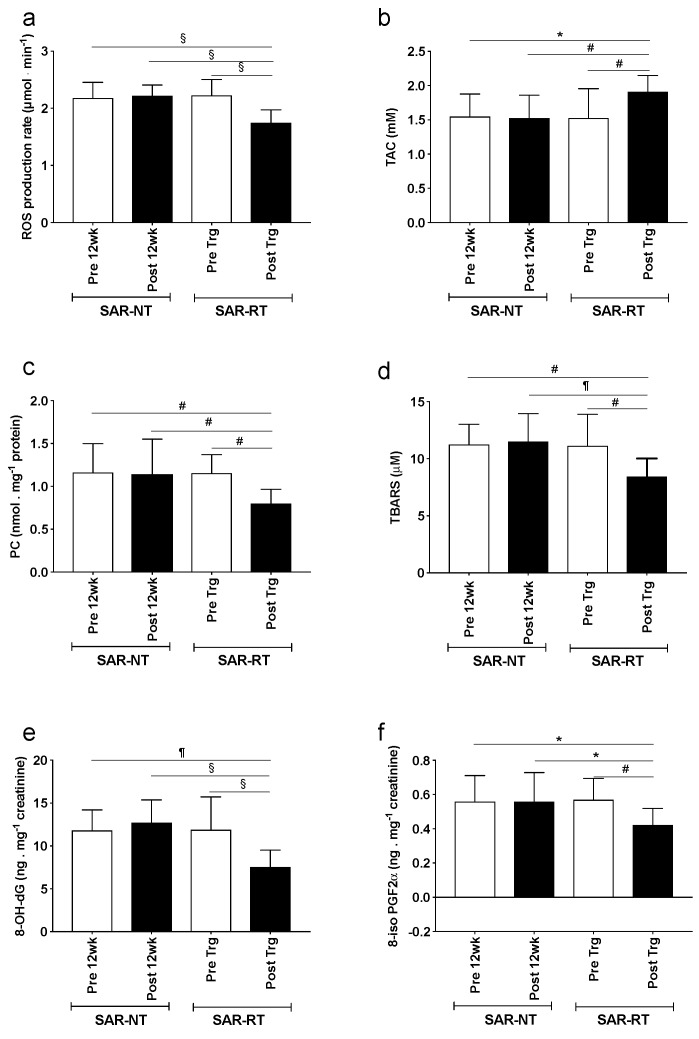
The effects of resistive training. The panel plots of: (**a**) ROS production rate (µmol·min^−1^), (**b**) total antioxidant capacity (TAC, mM), (**c**) PC (nmol·mg^−1^ protein), (**d**) TBARS (µM), (**e**) 8-OH-dG (ng·mg^−1^ creatinine), and (**f**) 8-iso-PGF2α (ng·mg^−1^ creatinine) recorded in sarcopenic participants acting as controls for 12 weeks (SAR-NT) and sarcopenic participants (SAR-RT) training for 12 weeks pre (empty bars), and post (filled bars) are shown. The data are presented as the mean ± SD. Significance of differences: *P* < 0.05 (*), *P* < 0.01 (#), *P* < 0.001 (¶), *P* < 0.0001 (§).

The OxS biomarkers determined from urine and blood samples in both SAR-RT and SAR-NT are shown in Figure 3. The ROS production rate in capillary blood (Figure 3a), TAC (Figure 3b), OxS biomarkers concentrations: PC (Figure 3c) and TBARS (Figure 3d) in plasma, 8-OH-dG (Figure 3e) and 8-iso-PGF2-α (Figure 3f) in urine assessed in sarcopenic participants before and after 12 weeks of training (SAR-RT) or control (SAR-NT) are displayed.

No statistical differences for any of the OxS variables were found between SAR-RT and SAR-NT at Pre. Moreover, no changes were observed for these markers between pre and post in SAR-NT. In SAR-RT, the ROS production rate decreased significantly from pre to post (21.2%, *P* < 0.001) reaching a value significantly lower (*P* < 0.001) than that observed in SAR-NT participants in both pre and post. Moreover, in SAR-RT all indexes of OxS damage were significantly reduced after training intervention. More specifically, PC decreased by 30.4% (*P* < 0.01), TBARS by 24.2% (*P* < 0.001), urinary 8-OH-dG by 36.7% (*P* < 0.001), and 8-iso-PGF2-α by 26.3% (*P* < 0.01) in Pre and Post, respectively. Conversely, plasma TAC significantly increased from 24.8% (*P* < 0.01) after RT training. The statistical differences (range 0.05 < *P* < 0.0001) for any of the OxS variables were found between SAR-RT recorded after RT and SAR-NT values assessed both at pre and post-12 weeks.

**Figure 4:**
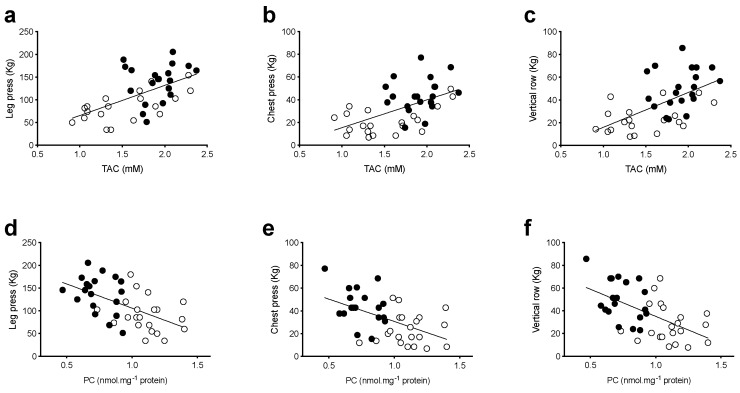
TAC and PC values versus muscle strength correlation at pre and post training. Multipanel plots of the relationships between individual total antioxidant capacity (TAC, mM) and protein carbonyls (PC, nmol·mg^−1^ protein) values measured in venous blood vs muscle strength (1RM, kg) evaluated as: (**a**) leg press, (**b**) chest press and (**c**) vertical row recorded pre (empty symbols) and post (full symbols) 12 weeks of progressive resistance training are shown. The linear regression fit (solid line) for each relationship is shown too. A significant positive relationship was observed between muscle strength at leg press (*P* = 0.0001, R^2^ = 0.33, (**a**)), chest press (*P* = 0.0003, R^2^ = 0.30, (**b**)), vertical row (*P* = 0.0001, R^2^= 0.34, (**c**)) recorded values and the correspondent TAC values. A significant inverse relationship was observed between muscle strength at leg press (*P* = 0.0002, R^2^ = 0.31, (**d**)), chest press (*P* = 0.0008, R^2^ = 0.27, (**e**)), vertical row (*P* = 0.0002, R^2^= 0.31, (**f**)) recorded values and the correspondent PC values.

Notably, a significant positive relationship was observed between individual muscle strength recorded values at leg press (*P* = 0.0001, R^2^ = 0.33, Figure 4a), chest press (*P* = 0.0003, R^2^ = 0.30, Figure 4b), vertical row (*P* = 0.0001, R^2^ = 0.34, Figure 4c) and the correspondent TAC values, suggesting a direct effect of RT training on antioxidant defenses. Otherwise, a significant inverse relationship was observed between muscle strength at leg press (*P* = 0.0002, R^2^ = 0.31, Figure 4d), chest press (*P* = 0.0008, R^2^ = 0.27, Figure 4e), vertical row (*P* = 0.0002, R^2^ = 0.31, Figure 4f) recorded values and the correspondent PC values.

## 4. Discussion

The main finding of this study is that in a population of healthy sarcopenic older individuals, a physical intervention based on moderate intensity resistance exercise leads to clear benefits at the molecular, structural and functional levels. The intervention used in this study, based on a 12-week RT at 60% 1RM with 3 × 14–16 reps, is effective for increasing muscle size and strength in a population of sarcopenic men and women aged > 65 years. Notably, highly significant gains (67–101%) in muscle strength (1RM) of muscle groups of the upper and lower limbs (chest press, vertical row, dumbbell lateral raise, and leg press), and in stair climbing power (~8%) were found after the 12-week RT intervention. These improvements in muscle function were associated with significant gains in muscle thickness, pennation angle and RF ACSA. Hence, these findings confirm that moderate intensity RT can be effective for inducing muscle growth and strength gains in older individuals. Although no change in skeletal muscle mass index (SMI) or SPPB score were found after training, it ought to be pointed out that in this study, SMI was assessed with BIA, whose reliability in detecting changes in muscle mass has been previously questioned [41].

None of these participants had reduced mobility as classified by the European Working Group on Sarcopenia in Older People (EWGSOP) definition, since all had a gait speed > 0.8 m·s^−1^, so they could not be defined sarcopenic according to the EWGSOP definition. Hence, it seems not surprising that these participants showed no change in SMI or SPPB in response to RT. Furthermore, on the basis of the new EWGSOP (EWGSOP2) definition [42], sarcopenia is classified as: (1) probable in the presence of a low muscle strength; (2) confirmed if in addition to criterion 1, low muscle quantity or quality are present; and (3) severe if in addition to criteria 1 and 2 there is also low physical performance. Thus, according to this latest definition of sarcopenia, none of the participants of the present study could be classified as severely sarcopenic.

Nevertheless, after the RT program, the number of elderly with type II sarcopenia (based on % SMI) was reduced by half. Moreover, a hypertrophic response to RT was detected after training, according to the high sensitivity and specificity of ultrasound in measuring changes in muscle size and architecture [43]. Therefore, given the significant gains in muscle function and in muscle size, it seems that the use of BIA and of the SPPB performance test are less sensitive for detecting changes in muscle mass and performance afforded by moderate intensity RT in type I sarcopenic (1) elderly.

Although our findings and those of previous studies [44,45] provide evidence that moderate to high intensity RT is effective for improving muscle mass, strength, and functional performance in healthy-elderly individuals, the molecular mechanisms underlying the positive effects of exercise in preventing age-related loss of muscle mass are still poorly understood. One mechanism may be related to the anti-oxidative benefits afforded by resistive exercise training.

The results of this study and those of previous literature [46] give support to the notion of ROS involvement in the etiology of the loss of muscle mass and strength and that the overproduction of oxidative damage may play a role in regulating intracellular signal transduction pathways, directly or indirectly involved in skeletal muscle atrophy and alterations of muscle contractility. Previous studies investigated the effects of RT on oxidative stress biomarkers, with contrasting findings [25]. In older adults, no changes in lipid peroxidation products and antioxidant capacity were reported [47]. On the contrary, others [48,49] showed that RT reduced the level of lipid peroxidation. Moreover, Parise and coworkers [50,51] showed that 14 weeks of RT increased the antioxidant mitochondrial enzymes and decreased the nucleic acid oxidation.

In line with previous reports [25,50,51], the changes in the oxidative stress balance in response to RT may have arisen from a decreased ROS production, due to an increased non-enzymatic antioxidant capacity as shown by the before/after training SAR-RT data comparison (Figure 3b). It seems noteworthy that the present changes in oxidative stress balance were produced by a RT intervention performed at considerably lower intensity than that used by others [47,52]. Hence, this study confirms that a significant reduction in oxidative stress is afforded by moderate intensity RT, in line with previous literature [25]. The net results of these changes (i.e., increase in total antioxidant capacity with consequent reduction of ROS production) led to an attenuation of the oxidative stress response (Figure 3c–f). Hence, one of the molecular mechanisms by which exercise counteracts the age-related loss of muscle mass may be the anti-oxidative action of exercise training, with the overexpression of the antioxidant system and reduced markers of oxidative damage.

Moreover, the results of the present study seem to confirm the key-role played by OxS on muscle force generating capacity. Indeed, TAC strongly correlated with the upper and lower limb muscle strength (Figure 4) and a reduction in ROS production rate, and OxS damaged biomarkers were observed after RT training. These modifications in OxS may represent a mechanistic basis for the improvement of muscle strength and the reduction in sarcopenia. Redox status is known to modify the ryanodine receptors (RyRs) embedded in the sarcoplasmic reticulum membrane, leading to failure of the excitation-contraction coupling [53]. Moreover, an OxS-mediated impairment of force generation through the interference with the cross-bridge cycling is possible, since contractile proteins have been shown to be redox-sensitive [54]. Hence, a better OxS balance may have improved the excitation-contraction coupling and thus cross-bridge cycling, resulting in increased force generation capacity.

A reduced protein activation may also be considered another possible mechanism related to the improved muscle force and changes observed in this study. Muscle mass is controlled by a dynamic balance of protein synthesis and degradation. A loss of muscle mass occurs when protein synthesis and degradation tip toward net degradation. The activity of the ubiquitin-proteasome pathway is transiently increased in response to decreased muscle use. The increases in the pathway activity via second messengers (i.e., ROS) are expected to stimulate both signaling events and general proteolysis, promoting the degradation of muscle proteins [55]. Therefore, lower levels of ROS production following resistive exercise training may slow the wasting of muscle mass by limiting the activation of protein degradation signaling.

Moreover, it has been found that protein carbonylation promotes cellular dysfunction and a decline in muscle fiber specific force [56] as also recorded by our results (Figure 4d–f). These data are in accord with Beltran Valls and coworkers [57] who reported that mitochondrial protein carbonylation increases moderately with age, and that this increase may influence skeletal muscle function.

Finally, this study demonstrated that 12 weeks of RT training reduced DNA damage (Figure 3e). These findings are different from those reported by Rall and coworkers [52]. The discrepancy in these findings can probably be related to the higher exercise intensity performed in the previous work (i.e., 80% 1-RM) compared to this study. Since DNA mutations have been shown to cause mitochondrial dysfunction and activate muscle cell apoptosis [9], these results highlight another potential benefit of the proposed low/moderate intensity RT.

Hence, our findings of a reduced ROS production, PC concentration, DNA damage and increased TAC value after the RT intervention strongly support the key role played by RT in reducing OxS in sarcopenic older individuals.

## 5. Limitations

The authors are aware that the experimental setting presented certain limitations. The food intake of the sarcopenic patients during the training intervention was not standardized, therefore, its contribution to the antioxidant capacity cannot be assessed. However, the participants were encouraged to maintain their regular activities of daily living and nutritional habits throughout the experimental period. The observed increase of TAC and decrease in OxS biomarkers are hardly attributable to a different antioxidant intake by food and drinks. Additionally, the biomarkers measured using blood and urine samples do not necessarily accurately reflect the redox status in skeletal muscle. Nevertheless, blood interacts with all organs and tissues and it is reasonable to assume that a systemic steady state level is reached in blood avoiding an invasive muscle biopsy. Moreover, the current study is based on a small sample of participants, which is not enough to allow a gender difference analysis.

## 6. Conclusions

The present study provides evidence that a moderate-intensity RT program can be highly effective for combating sarcopenia in older individuals by inducing significant gains in muscle mass and strength, while minimizing ROS production and oxidative stress. These findings are consistent with previous observations that moderate-intensity high-volume resistance exercise can overcome anabolic resistance and maximise protein synthesis, in older adults.

## Figures and Tables

**Table 1:** Features of the groups of participants.

	SAR-NT		SAR-RT	
	n = 15 9 F/6 M		n = 20 10 F/10 M	
	Pre 12 Weeks	Post 12 Weeks	Pre Training	Post Training
	Mean ± SD	Mean ± SD	Mean ± SD	Mean ± SD
**Age (years)**	71.7 ± 3.4	71.7 ± 3.4	73.0 ± 5.5	73.0 ± 5.5
**Body Mass (kg)**	69.8 ± 15.0	70.2 ± 15.4	76.3 ± 16	75.7 ± 16.8
**Stature (m)**	1.62 ± 0.1	1.62 ± 0.1	1.65 ± 0.1	1.65 ± 0.1
**BMI (kg m^−2^)**	26.6 ± 3.5	26.8 ± 3.6	27.7 ± 4.4	27.5 ± 4.7
**Skeletal Muscle Mass (kg)**	20.8 ± 6.3	21.2 ± 5.9	22.5 ± 6.3	22.9 ± 6.4
**SMI%**	29.6 ± 3.8	29.8 ± 3.6	29.4 ± 4.7	30.2 ± 4.2 ^#^
**Handgrip (kg)**	27.8 ± 9.4	29.4 ± 9.6	32.4 ± 10.7	30.9 ± 10.6
**SPPB Score**	11.7 ± 0.4	11.6 ± 0.4	11.0 ± 1.8	11.2 ± 1.2
**Get-Up and go Score**	n = 15 freely mobile	n = 15 freely mobile	n = 19 freely mobile n = 1 mostly independent	n = 20 freely mobile
**Stair Climbing (W)**	256.1 ± 87.6	262.4 ± 84.5	299.8 ± 114.0	322.8 ± 148.2 ^##^
**Classification**				
**Non sarcopenic**	n = 0	n = 0	n = 0	n = 3
**Class I sarcopenia**	n = 14	n = 14	n = 14	n = 14
**Class II sarcopenia**	n = 1	n = 1	n = 6	n = 3

The data are presented as mean ± standard deviation (SD). M, males; F, females; SAR-NT, age-matched sarcopenic controls; SAR-RT, sarcopenic training for 12 weeks; BMI, body mass index; SMI%, skeletal mass index as percentage; SPPB, short physical performance battery test. Significance: ^#^ statistically different from pre- with *P* < 0.05; ^##^ statistically different from pre- with *P* < 0.01.

**Table 2:** Changes in muscle morphology between pre and post-12 weeks in both sarcopenic control (SAR-NT) and sarcopenic resistance training (SAR-RT) groups.

	SAR-NT			SAR-RT				
Muscle Morphology	Pre 12 Weeks	Post 12 Weeks	P	Pre Training	Post Training	Difference %	P	MDC_95_%
VL—PA (°)	15.7 ± 2.5	15.6 ± 2.3	0.092	13.5 ± 3.1	15.3 ± 2.8	+13.4%	<0.001	9.6%
VL—MT (cm)	1.6 ± 0.2	1.7 ± 0.3	0.202	1.7 ± 0.3	1.8 ± 0.4	+5.5%	0.002	4.9%
RF—ACSA (cm^2^)	3.7 ± 1.1	3.7 ± 1.1	0.226	4.0 ± 1.3	4.5 ± 1.4	+14.5%	<0.001	10.0%
EF—MT (cm)	2.4 ± 0.5	2.4 ± 0.4	0.123	2.6 ± 0.6	2.8 ± 0.9	+10.4%	<0.001	8.3%

The data are presented as the mean ± SD. SAR-NT, age-matched sarcopenic controls pre- and post-12 weeks (12 week); SAR-RT, sarcopenic group undergoing 12 week of progressive resistance training; VL, vastus lateralis muscle; RF, rectus femoris muscle; EF, elbow flexors muscles; PA, pennation angle; MT, muscle thickness; ACSA, anatomical cross-sectional area; MDC_95_%, minimum detectable change at 95% confidence as percentage; P, *P*-value.

**Table 3:** Differences in muscle strength between pre and post-12 weeks of progressive resistance training in the sarcopenic training group (SAR-RT).

1 RM (kg)	Pre	Post	Difference	P	MDC_95_%
Chest press	22.2 ± 12.3	44.6 ± 15.4	+101.0%	<0.001	15.0%
Leg press	90.0 ± 32.2	167.2 ± 87.3	+85.8%	<0.001	9.4%
Vertical row	26.5 ± 15.5	50.2 ± 17.4	+88.7%	<0.001	15.6%
Lateral rise	2.6 ± 1.5	4.3 ± 1.8	+66.6%	<0.001	39.3%

The data are presented as the mean ± SD. 1RM, one-repetition maximum; MDC_95_%, minimum detectable change at 95% confidence as percentage; P, *P*-value.

## Abbreviations

RT	resistive training
SAR	sarcopenic elders
SMI	skeletal muscle index
VL	vastus lateralis
EF	elbow flexors
MT	muscle thickness
PA	pennation angle
RF	rectus femoris
ACSA	anatomical cross-sectional area
EPR	electron paramagnetic resonance
ROS	reactive oxygen species
TAC	total Antioxidant Capacity
PC	protein carbonyls
TBARS	thiobarbituric acid-reactive substances
8-iso-PGF2-α-	8-isoprostane
8-OH-dG	8-OH-2-deoxyguanosine
OxS	markers of oxidative stress/damage
